# The association between lower limb fractures and weight gain in adults: a prospective analysis of body mass index trends

**DOI:** 10.1007/s00590-024-03832-x

**Published:** 2024-02-01

**Authors:** Ali Lari, Abdullah Haidar, Hussain Mohammad, Mohammad Makhseed, Mejbel Alajmi, Retaj Bahbahani, Majed Almutairi, Naser Alnusif, Eisa Lari

**Affiliations:** 1 Department of Orthopedic Surgery, AlRazi National Orthopedic Hospital, Kuwait City, Kuwait; 2 Department of Orthopedic Surgery, Mubarak Al Kabeer Hospital, Kuwait City, Kuwait; 3 Department of Orthopedic Surgery, Jaber Al Ahmad Hospital, Kuwait City, Kuwait; 4 School of Medicine, Kuwait University, Kuwait City, Kuwait; 5 Department of Surgery, Jaber Al Ahmad Hospital, Kuwait City, Kuwait

**Keywords:** Fractures, Lower limb Trauma, Body mass index, Weight gain, Obesity

## Abstract

**Purpose:**

Despite understanding the connection between obesity and fracture risk, there is limited research on the implications of lower limb fractures on subsequent changes in body mass index (BMI). Our study aimed to assess the impact of lower limb fractures on BMI alterations over an 18-month period.

**Methods:**

A multi-center, prospective cohort study was conducted between January 2021 to June 2023, involving 494 adults with lower limb fractures. Participants were recruited within 2 weeks post-injury and were assessed for demographics, injury details, and weight at seven distinct time points. By 18 months, the primary outcome was the mean weight gain.

**Results:**

The average age of the participants was 39 (± 12.7) with a baseline weight and BMI of 80.4 kg and 27.6, respectively. At the 18-month follow-up, 75% of patients experienced an average weight increase in 4 kg (± 5.39 kg), equating to a BMI rise of 1.39 (± 1.88). Most patients attributed weight changes to their injury, with nearly half expressing distress from their weight change. Only 37% believed that they had resumed their previous activity levels by the final follow-up. Approximately 31% of the patients sought some form of external weight management care in the form of nutritionist advice, training programs, medication and weight management procedures.

**Conclusions:**

Lower limb fractures significantly affect weight gain over an 18-month period, with substantial psychological and physical consequences. Healthcare providers should anticipate potential weight gain post-fracture and incorporate strategies addressing both physical and mental aspects of rehabilitation to enhance recovery outcomes. Early and even immediate weight bearing may play a pivotal role in mitigating weight changes and returning the patient to their previous level of activity. Further detailed studies focusing on different fractures and postoperative interventions are recommended.

## Introduction

Lower extremity fractures are ubiquitous in orthopedic practice, often resulting in considerable morbidity. There are numerous mechanisms of injuries, ranging from both low and high energy trauma [1, 2]. The ramifications of such fractures extend far beyond the acute physical impairment and often necessitate some sort of intervention. Frequently, there is significant disruption to the overall health and well-being of the individuals involved [3, 4]

The relationship between obesity and fracture risk has been a subject of substantial interest within the scientific community. Initially, obesity was believed to confer a protective effect against fractures [5]. However, more recent evidence refutes this notion, suggesting that obesity, in fact, increases the risk of fractures [6–8]. While these studies establish a connection between weight and fracture risk, no research has yet assessed the potential impact of a lower limb fracture on subsequent changes in body mass index (BMI). This is a particularly interesting area to explore as injury and obesity carry compounding negative effects. Physiological and psychological responses to such injuries may affect eating habits, physical activity and in turn, lead to poor weight management.

As such, it is crucial to understand the implications of lower limb fractures on BMI over recovery period. When considering the high incidence of lower limb fractures, any influence on BMI may present significant implications to public health. This carries more concern in light of the rising rates of obesity and the associated complications worldwide. The global prevalence of obesity is increasing, and in some countries surpasses 50%, with no indication of a decline in this upward trend observed over recent decades [9]. Moreover, understanding the potential for increased obesity risk following lower limb fractures could enable healthcare practitioners to tailor rehabilitation and recovery strategies.

Despite the importance of this issue, there is a notable scarcity of research investigating the effects of lower limb fractures on BMI. While previous studies have examined the impact of fractures on overall quality of life [10, 11], none have delved into the change in BMI following a fracture. Our study aims to bridge this gap with an initial exploratory investigation into the effect of lower limb fractures on BMI.

## Methods

This study was a multi-center, prospective cohort study conducted in one tertiary orthopedic hospital and two general hospitals from January 2021 to June 2023. Prior to their participation in the study, informed consent was obtained from eligible patients. Ethical approval was obtained from our institution (UID: 2021/1902).

### Study population

The eligible patients, aged between 18 and 65, had sustained lower limb fractures. Inclusion in the study required recruitment within the first 2 weeks post-injury. Patients were either enlisted from the outpatient department during their initial visit or as inpatients. The exclusion criteria included: presence of upper extremity fractures, pelvis and acetabulum fractures, inability to walk independently prior to injury, and significant medical comorbidities that could affect weight, such as diabetes, thyroid disorders, psychiatric disorders, eating disorders, cardiovascular disease, and chronic kidney disease. Additionally, those who had undergone weight management procedures like intragastric balloons or bariatric surgery within the follow-up period were also excluded. A total of 158 patients were excluded from the final analysis: 145 due to loss of follow-up and incomplete data, primarily because they had traveled abroad, and 13 due to weight management procedures performed before the 18-month follow-up, including intragastric balloons and sleeve gastrectomy.

### Outcome measures

All patients were evaluated for baseline demographic and injury characteristics including age, sex, occupational activity, type of injury, and treatment method. We also assessed the presence of immobilization post-injury, presence of multiple fractures, baseline weight, and height. Weight was measured at seven different time points. At the final 18-month follow-up, we asked patients various questions related to their weight, activity levels, and weight management efforts. Occupational activity was defined based on the accelerometry-based classification used by the National Health and Nutrition Examination Survey [12].

Weight was measured using the in-hospital scales during outpatient follow-ups or as inpatients for those requiring prolonged in-hospital stays. If weight was not measured during the follow-up, patients were contacted by phone and asked to weigh themselves at the appropriate time point. The consistency of self-reported weight with measured weight was investigated by both Wing et al. and Stunkard et al., with both reporting high levels of reliability and accuracy [13, 14].

### Statistical analysis

Statistical analysis was performed using R v 3.6.3. Counts and percentages were used to summarize categorical variables. The mean ± standard deviation and the median/interquartile range (IQR) were used for continuous normal and non-normal variables, respectively. Linear regression analysis was used to assess factors associated with absolute weight gain at 1 year and 18 months. Hypothesis testing was performed at 5% level of significance. In this prospective cohort study, the primary outcome was the mean weight gain over an 18-month period following lower limb fractures.

Given the nature of the study, we estimated the standard deviation (*σ*) of weight gain to be 4 kg. Our goal was to estimate the true population mean weight gain with a precision of ± 1 kg, defined as our acceptable margin of error (*ε*). We also sought to ensure 95% confidence in our estimate, corresponding to a *Z*-value of 1.96 for a two-tailed test. We adjusted for an anticipated 40% loss to follow, giving an adjusted sample size of 410. The high rate of follow-up was based on a pilot study conducted prior to the initiation of this study. Further, the patient population involved consisted of many expatriate labor workers who have high rates of travel back to homeland after injury.

## Results

The baseline demographics and injury characteristics are shown in Table 1. We included a total of 494 patients, with a mean age of 39 (± 12.7), in our final analysis. The baseline weight and BMI were 80.4 kg and 27.6, respectively. Fractures were predominantly foot and ankle injuries (64.8%), trailed by tibial and femoral shaft fractures (15%) and peri-articular knee injuries (12.3%). The majority of patients underwent open reduction internal fixation (ORIF) (69%), while immobilization using splints, plaster of Paris, and braces was utilized in 45% of participants.

**Table 1:** Baseline demographics and injury characteristics

	[ALL]
	*N* = 494 Mean (SD)
Age	39.0 (12.7)
**Sex**	
F	81 (16.4%)
M	413 (83.6%)
Height (cm)	171 (8.50)
Baseline weight	80.4 (16.3)
Baseline BMI	27.6 (5.39)
**Fracture location**	
Ankle	241 (48.8%)
Calcaneus and tarsal bones	42 (8.50%)
Femur	62 (12.6%)
Metatarsal	36 (7.29%)
Patella	15 (3.04%)
Tibia	98 (19.8%)
**Injured side**	
Both limbs	35 (7.09%)
Lt	243 (49.2%)
Rt	216 (43.7%)
Smoker	132 (26.7%)
Hypertension	47 (9.51%)
**Occupational activity (OA)**	
High OA	94 (19.0%)
Intermediate OA	89 (18.0%)
Low OA	311 (63.0%)
**Regional classification**	
Foot and ankle	320 (64.8%)
Hip	36 (7.29%)
Knee	61 (12.3%)
Shaft	77 (15.6%)
Immobilization	225 (45.5%)
Multiple fractures	59 (11.9%)
**Treatment**	
External—fixation	25 (5.20%)
Intramedullary nail	76 (15.5%)
Non-operative	49 (10.0%)
ORIF	340 (69.3%)

Data were summarized using counts and percentages

**Fig. 1:**
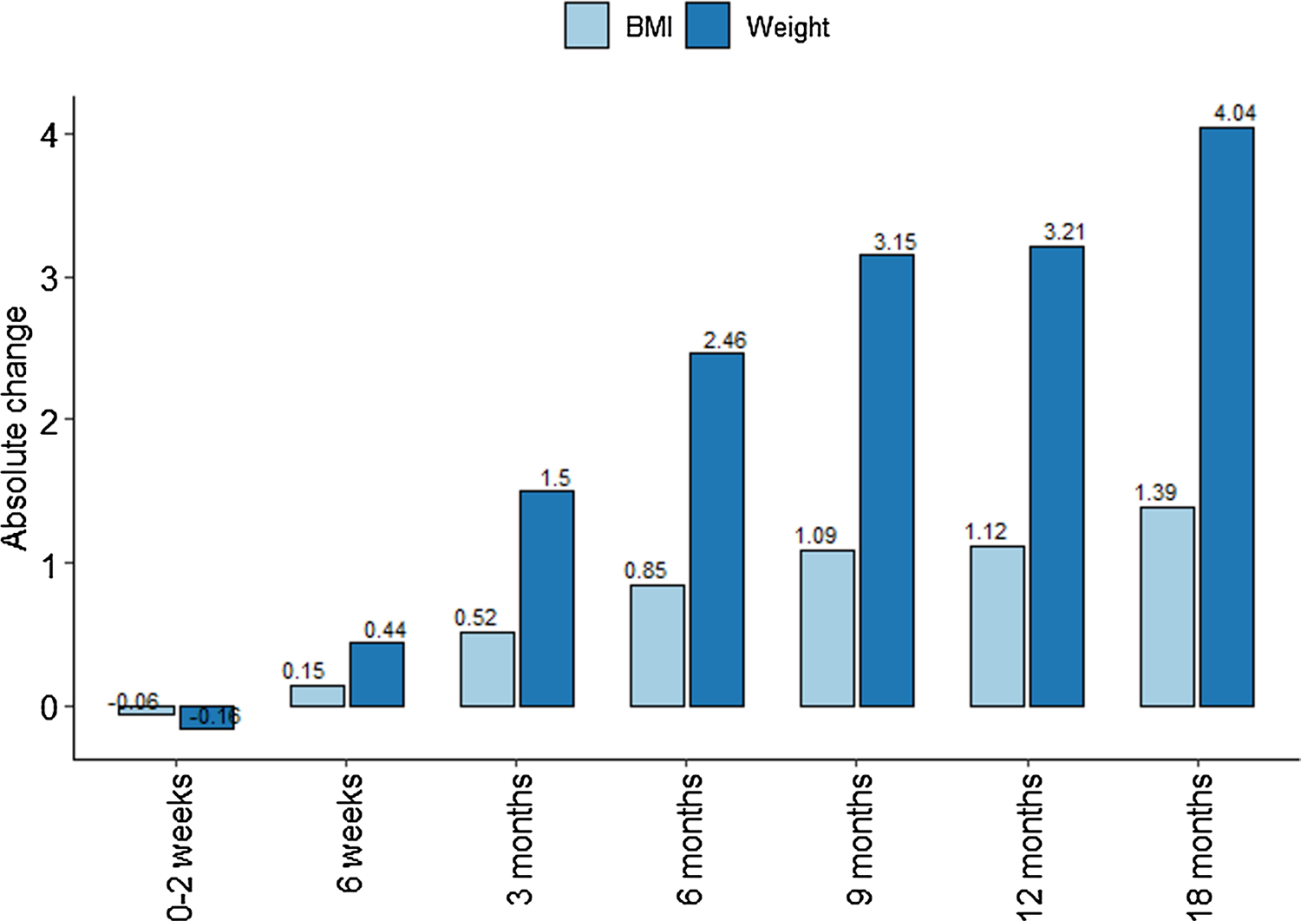
A chart summarizing the absolute weight gain and body mass index across different time points

Overall, 75% of the sample population experienced weight gain by the 18-month follow-up (Table 2). A weight gain trend was visible after the 6-week follow-up. The initial visit did not show any significant weight gain, instead some weight loss was noted compared to the baseline weight. By 18 months, an average weight increase in 4 kg and a BMI increase in 1.39 kg/m^2^ were observed (Fig. 1).

**Table 2:** Average absolute change in (A) weight and (B) BMI at different time points

Time point and variable	Mean	SD	Relative weight gain (%)	*N* (%) of patients who gained weight
Weight gain 0–2 weeks	− 0.16	1.94	− 0.15	201 (40.7%)
Weight gain 6 weeks	0.44	2.56	0.60	247 (50.0%)
Weight gain 3 months	1.50	3.60	1.97	305 (61.7%)
Weight gain 6 months	2.46	4.19	3.21	327 (66.2%)
Weight gain 9 months	3.15	4.71	4.06	352 (71.3%)
Weight gain 12 months	3.21	4.66	4.13	361 (73.1%)
Weight gain 18 months	4.04	5.39	5.19	372 (75.3%)
BMI gain 0–2 weeks	− 0.06	0.66		
BMI gain 6 weeks	0.15	0.88		
BMI gain 3 months	0.52	1.25		
BMI gain 6 months	0.85	1.46		
BMI gain 9 months	1.09	1.63		
BMI gain 12 months	1.12	1.62		
BMI gain 18 months	1.39	1.88

Our analysis did not find any significant associations between fracture type and weight gain (Table 3). The smallest change in weight was seen in patients with calcaneus fractures, while the highest weight gain was observed in patients with metatarsal fractures, followed by patella and femur fractures. However, these findings were not statistically significant.

**Table 3:** Association between fracture type and weight gain

Absolute weight gain (kg)	Ankle	Calcaneus	Femur	Metatarsal	Patella	Tibia	*P* value
	*N* = 241	*N* = 42	*N* = 62	*N* = 36	*N* = 15	*N* = 98	
WT 0–2 weeks	− 0.16 (1.78)	− 0.43 (1.48)	− 0.03 (2.39)	− 0.33 (2.12)	0.27 (3.56)	− 0.14 (1.79)	0.837
WT 6 weeks	0.38 (2.32)	0.10 (2.28)	0.63 (2.81)	0.69 (2.98)	0.33 (4.15)	0.53 (2.63)	0.886
WT 3 months	1.41 (3.54)	0.62 (2.80)	1.81 (3.55)	2.08 (3.80)	1.33 (5.16)	1.69 (3.75)	0.499
WT 6 months	2.29 (4.27)	1.67 (3.21)	3.11 (4.03)	3.19 (4.57)	1.93 (4.95)	2.60 (4.20)	0.432
WT 9 months	3.11 (4.90)	2.29 (4.15)	3.68 (4.66)	3.56 (4.22)	3.33 (4.82)	3.11 (4.71)	0.778
WT 12 months	3.44 (4.55)	1.67 (4.75)	3.68 (4.44)	3.42 (5.35)	3.67 (5.97)	2.87 (4.49)	0.257
WT 18 months	4.07 (5.11)	2.05 (5.20)	4.42 (5.72)	5.17 (6.00)	4.47 (6.32)	4.13 (5.48)	0.171

Data were summarized using mean (SD), and Analysis was performed using one-way ANOVA

Only 16.4% of the patients discussed their weight concerns with their treating physicians. Nearly half (44.7%) of the patients reported distress as a result of their weight change. Furthermore, only 37% of patients believed that they had returned to their previous activity levels at the final follow-up. Approximately 31% of the patient sought some form of external weight management care in the form of nutritionist advice, training programs, medication and weight management procedures (Table 4).

**Table 4:** Survey-based responses on weight management and activity levels

	[ALL]
	*N* = 494
Physical activity	
Back to baseline	187 (37.9%)
Reduced since fracture	307 (62.1%)
Self—diet/exercise	174 (35.2%)
WM treatment (nutrition/trainer/program)	92 (18.6%)
WM medication	40 (8.10%)
WM procedure done/planned (after 18 months)	24 (4.86%)
Direct attribution to fracture	229 (46.4%)
Weight changes caused you distress	221 (44.7%)
Was weight discussed with treating doctor	81 (16.4%)

## Discussion

Considerable evidence establishes an association between obesity and fracture risk, with research primarily focusing on the vulnerability of individuals already classified as obese [15–17]. Our initial study, examining the effect of lower extremity fractures on BMI changes, uncovered several key findings. These pertain to weight gain, BMI alterations, the psychological consequences of shifting weight, and the ensuing impacts on physical activity levels.

The key finding in this research is a significant increase in patients' weight. The initial 6 weeks showed insignificant weight changes; thus, no definitive conclusions could be drawn from such a short period. However, an observable weight increase appeared at the 18-month follow-up, where 75% of patients saw an average 4 kg (± 5.39 kg) increase, corresponding to a 1.39 (± 1.88) increase in BMI. In contrast, Tucker et al.'s research on the 10-year weight variances in over 13,000 adults reported an average 4.2 (± 0.2) kg increase. This comparison underscores two themes: the pace of weight gain and the wider span of weight variation following lower limb fractures.

The observed weight gain may stem from multiple behavioral changes, such as reduced physical activity levels and potential dietary modifications. Impediments like diminished functional capacity and emotional disruptions may persist beyond fracture healing [1]. This study emphasized the link between reduced physical activity and obesity [18, 19]. The weight gain could have diverse repercussions, including exacerbating pre-existing medical conditions, diminishing overall well-being, and potential mental health impacts. This emphasizes the importance of considering the patient's holistic health, not merely the objective fracture healing measures.

In this research, we noted various fracture types, but no meaningful conclusions could be drawn about weight gain variations among different groups. Patients underwent distinct procedures and followed different postoperative protocols based on their injuries, possibly influencing their rehabilitation progress. Nevertheless, the study aimed to uncover the overarching link between fractures and excessive weight gain.

Our study highlights the personal measures taken by patients to manage their weight. The majority attributed their weight changes to their injuries, causing significant distress, and reported reduced activity levels in the final follow-up. Additionally, patients took various measures, such as dietary adjustments, participation in weight management programs, use of weight management medication, and even contemplated weight management procedures. Despite these efforts, weight changes distressed 44.7% of the study participants. Yet, only 16% reported discussing this issue with their treating practitioners. These results align with prior research on weight gain's psychological impact [20]

Post-fracture physical activity is critical to recovery from lower limb fractures. However, only a third of the patients perceived their activity levels to be normalized. Previous studies show that patients are unlikely to regain their pre-fracture physical health [21]. This emphasizes the importance of setting realistic expectations for patients about regaining pre-injury activity levels [22].

Addressing potential weight gain may be challenging due to the paucity of data in orthopedic trauma, particularly because orthopedic surgeons may not be deeply versed in weight management strategies. However, taking cues from obesity research and actively involving patients in formulating strategies to mitigate this risk is prudent. Although patients play a large role in managing their own weight, we would stress that the surgeon’s input and early rehabilitation may be key to reducing the likelihood of significant weight gain.

Identifying patients at higher risk are perhaps the first step. Including those with a propensity for immobilization, chronic pain, extended recovery periods, existing overweight issues, or a generally sedentary lifestyle [23, 24]. Despite limited evidence in this area, a systematic approach is necessary to pinpoint patients in need of closer monitoring. Once identified, patient education becomes essential, setting expectations for potential challenges related to weight and mobility.

The next phase involves developing strategies to counteract weight gain, which may include collaboration with nutritionists and occupational therapists [25, 26]. Occupational therapists can offer adaptive strategies to remain active during limited mobility, while nutritionists can provide tailored dietary guidance to meet the unique needs of each recovering patient, both have been shown to have a significant role in reducing weight [25, 26]. Notably, many patients already sought nutritional advice independently, suggesting a gap in standard care practices. A comprehensive approach that addresses both physical and mental health is beneficial, as patients frequently experience psychological distress post-injury [27]. This distress can lead to diminished motivation and a decrease in adherence to therapeutic and dietary regimens, underscoring the need for strong morale.

Immobilization and splinting are still often seen for surgical fixation and has perhaps been anecdotally adopted into practice despite emerging evidence that has consistently supported the benefits of avoiding immobilization and promoting early mobilization [28, 29]. Similarly, casts and braces, while beneficial in select cases, may not be as effective as previously thought in the context of fracture healing and protection of fixation [30]. Despite the evidence, surgeons in good intent still carry some reluctance in allowing immediate or full weight bearing, leading to a counterproductive rehabilitation [29, 31]

Patients who are already facing obesity or overweight challenges may find the inclusion of a bariatric specialist in their treatment beneficial, especially if their injury exacerbates existing weight management difficulties. Another consideration is sarcopenic obesity, characterized by the loss of skeletal muscle and fat gain, a condition that complicates recovery and can lead to prolonged weakness that may take years to recover. This subset of patients requires intensive physical therapy and may even benefit from electrostimulation to regain muscle strength [32, 33]. As such, early involvement of physiotherapists is essential, especially for the elderly population.

While weight gain from reduced mobility may seem predictable, our study substantiates this notion with solid evidence. The research was limited by its general sampling approach and the variety of fractures and interventions examined. Furthermore, we did not explore postoperative regimens in detail, given the technical challenges of considering surgeon preferences and recruitment strategy. We prioritized the endpoint as weight gain beyond the typical fracture recovery period, where postoperative regimens might be less relevant.

## Conclusion

Lower limb fractures result in considerable weight gain over 18 months. Patients often report reduced activity and distress due to weight changes. Practitioners should consider the effects of potential weight gain on physical and mental rehabilitation in their efforts to optimize recovery. Strategies to manage weight gain during the recovery period are essential and early or even immediate weight bearing may play a pivotal role in mitigating weight changes. Future studies should scrutinize the impacts of various fractures, postoperative interventions, and regimens on body mass index more closely.
